# Successful management of mucinous ovarian cancer by conservative surgery in week 6 of pregnancy: case report and literature review

**DOI:** 10.1007/s00404-012-2490-4

**Published:** 2012-08-01

**Authors:** Shan-yang He, Hong-wei Shen, Lin Xu, Xiao-li Li, Shu-zhong Yao

**Affiliations:** 1 Department of Obstetrics and Gynecology, The First Affiliated Hospital, Sun Yat-sen University, Guangzhou, Guangdong 510700 People’s Republic of China; 2 Department of Microbiology, Zhongshan School of Medicine, Sun Yat-sen University, Guangzhou, Guangdong 510080 People’s Republic of China; 3 Department of Obstetrics and Gynecology, Memory of HeXiang Hospital, Panyu, Guangzhou, Guangdong 511400 People’s Republic of China

**Keywords:** Pregnancy, Ovarian cancer, Cytoreductive surgery, Chemotherapy

## Abstract

**Purpose:**

The management of ovarian cancer during pregnancy is still a big challenge, mostly due to the reciprocal impacts between cancer and pregnancy. The objective of this article is to present a rare case of maternal ovarian adenocarcinoma and review published similar cases about this clinical condition.

**Materials and methods:**

Here we report a rare case of maternal ovarian adenocarcinoma detected during gestational week 6, with good pregnancy outcome treated with conservative surgery.

**Results and discussion:**

A case of maternal ovarian adenocarcinoma (stage I) was detected in week 6 of pregnancy receiving conservative surgery without chemotherapy. In week 39 of pregnancy, due to relapse of the cancer, the patient underwent excision of the isolated tumor, and gave birth to a healthy baby through cesarian section. After that, the patient received cytoreductive surgery associated with six chemotherapy. The patient was finally diagnosed as epithelial ovarian cancer stage IIIC, and had survived more than 5 years without relapse. The successful experience from this case suggested that pregnancy complicated with early ovarian cancer receiving conservative surgery could continue to pregnancy and the effect of cesarian section followed with cytoreductive surgery associated with six chemotherapy at full term was still satisfied.

## Introduction

Ovarian cancer is the second most frequent gynecological cancer diagnosed during pregnancy, exceeded only by cervical carcinoma. Even so, ovarian cancer during pregnancy is rare, with an estimated incidence of 1:10,000 to 1:50,000 [1–3]. Among all the malignant ovarian tumors diagnosed during pregnancy, the germ cell tumor is reportedly the most prevalent, and epithelial ovarian cancer accounts for 20 % of all ovarian cancers [4–6]. Epithelial ovarian cancer affects predominantly perimenopausal and postmenopausal women. Ovarian adenocarcinoma, a subtype of epithelial ovarian cancer, is rarely detected during pregnancy in young women. The frequency of maternal epithelial ovarian cancer is likely to increase because of the increasing number of women who postpone childbearing [3, 5].

The clinical outcome of patients with epithelial ovarian cancer is not affected by pregnancy. The same surgical staging procedures are recommended for patients with or without pregnancy [2–4]. This cancer remains a significant clinical challenge, however, because ovarian carcinoma and the treatment for it during pregnancy affect not only the pregnant woman but also the fetus [4–6]. Although cases have been reported anecdotally, there have been no large-scale randomized trials or cohort studies because of the low incidence of ovarian cancer during pregnancy [4–6]. Case reports, however, provide important information on the management of these cancers [5]. We report a case of maternal ovarian adenocarcinoma detected during gestational week 6. The patient was treated with conservative surgery, without chemotherapy, and gave birth to a healthy baby during week 39 of the pregnancy.

## Case report

Prior written consent of the patient for the use of these clinical materials for research purposes, and approval from the Institutional Ethical Board (IRB) in the First Affiliated Hospital of Sun Yat-sen University were obtained. The patient was a 25-year-old woman who underwent abdominal ultrasonography (US) because of severe low quadrant pain that had lasted for 12 h during gestational week 6. The US showed a cystic solid mass measuring 9 × 6 cm with a small amount of blood from the left adnexa. No ascites was found. An emergent laparotomy was performed with a suspicion of mass torsion. Five serum tumor markers were measured preoperatively: α-fetoprotein (AFP), cancer antigen (CA)199, carcinoembryonic antigen (CEA), CA153, and CA125. CA125 was found to be abnormally elevated to 121 U/ml.

Laparotomy confirmed the presence of a left ovarian cyst with a maximum diameter of 9 cm and one round of torsion. It had a smooth, black surface without excrescences, and its section had no papillary proliferations. A right ovarian complex mass of 3 × 2 cm was also found. The left mass was managed by unilateral salpingo-oophorectomy. The right mass was removed by cystectomy. Both masses were completely removed, leaving an intact membrane. No ascitic fluid was found in the abdominal cavity during the procedure. The omentum, liver, and spleen were normal. No intraoperative peritoneal implant or suspicious areas for malignancy were found; so multiple peritoneal biopsies for surgical staging was not given. Peritoneal washing during her emergency laparotomy and intraoperative frozen section was not performed. The reason is that the junior doctors in emergency surgery believed the twisted mass was benign because the gross mass surface has no excrescences and was smooth. Another reason is that nodular necrotic vascular mass section has no papillary proliferations.

**Fig. 1:**
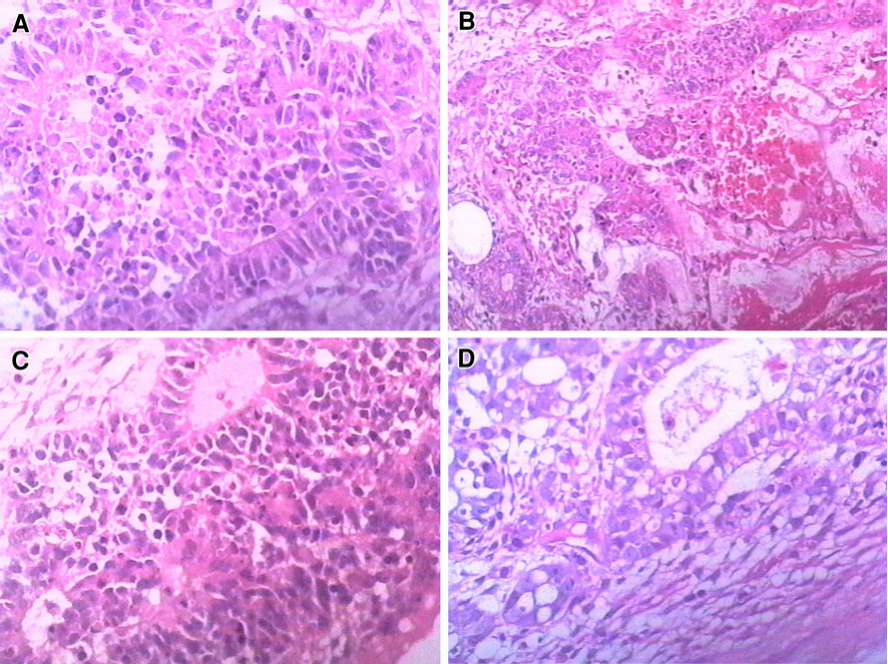
The pathological examination of the primary ovarian adenocarcinoma from a 25-year-old woman during gestational week 6. **a** Histological analysis showed highly differentiated mucinous cystadenocarcinoma grade I. Atypic glands arranged in dense and cancer cell with large and deeply stained nuclei arranged in disorder. HE staining ×400; **b** local hemorrhage of the ovarian adenocarcinoma, HE staining ×200; **c** local necrosis of the ovarian adenocarcinoma, HE staining ×400; **d** the intact membrane can be observed at the edge of the tumor. HE staining ×400

Histological analysis of the left mass showed solid and cystic areas with regions of hemorrhage and necrosis (Fig. 1a−c). It confirmed the diagnosis of mucinous cystadenocarcinoma grade I with intact membrane (Fig. 1d). The right mass was filled with grease and hair, and was diagnosed as a mature teratoma. Serum tumor markers including AFP, CA199, CEA, CA153, and CA125 were measured on postoperative day (POD) 3. The maternal serum CA125 had dropped from 121 U/ml preoperatively to 93 U/ml, but the other markers showed no obvious changes.

After extensive counseling by gynecological oncologists and pediatricians, the patient expressed a strong desire to continue the pregnancy and refused another operation for staging and decisions about chemotherapy. The patient left the hospital on POD 7 with a diagnosis of International Federation of Gynecologists and Obstetricians (FIGO) stage IA carcinoma (at least).

The patient presented again at 39^+1^ weeks of gestation with abdominal bloating and dyspepsia that had been present for about 7 days. US identified a normal fetus in the uterus. A complex mass measuring 12 × 11 cm and rich with blood was found between the bladder and uterus. It appeared to be predominantly cystic although there were some solid parts and papillary areas. These features suggested relapse of the ovarian adenocarcinoma. The maternal serum CA125 and AFP levels were elevated to 381 U/ml and 3,810 ng/ml, respectively.

The patient underwent excision of the isolated tumor with cesarean section at 39^+1^ weeks of gestation. Cytoreductive surgery was then performed, including total hysterectomy, right salpingo-oophorectomy, complete omentectomy, and pelvic and para-aortic lymph node resection.

The baby weighed 3,000 g and had an Apgar score of 10 at 1 min and a score of 10 at 5 min. US examinations of the baby at 1 month showed normal brain, liver, spleen and kidneys.

**Fig. 2:**
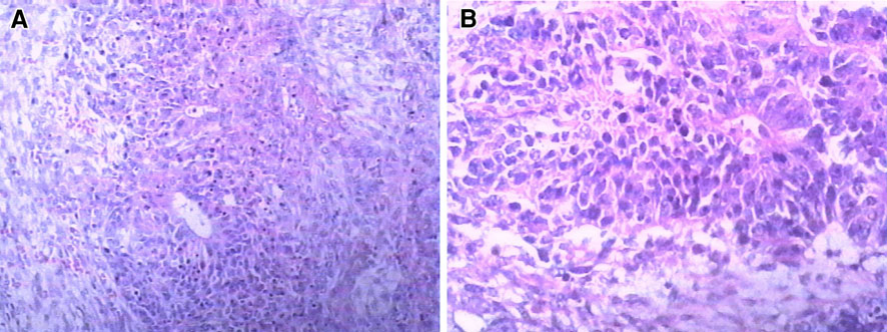
The abdominal metastatic ovarian adenocarcinoma at 39 + 1 weeks of gestation. Histological analysis showed metastatic moderately differentiated ovarian adenocarcinoma. Atypic glands were very few or almost none and small tumor cell with mitotic figures arranged in nests. **a** HE staining ×200; **b** HE staining ×400

Histopathological diagnosis of the tumor confirmed isolated metastatic ovarian cystadenocarcinoma. The lymph nodes, uterus, right adnexa, omentectomy and placenta were negative for metastasis (Fig. 2). The patient received six courses of cisplatinum 75 mg/m^2^ and docetaxel 75 mg/m^2^ every 4 weeks after surgery. The AFP and CA125 levels were normal on POD 28 and after the third cycle of chemotherapy, respectively. The patient and her baby were followed up for more than 5 years, during which time the serum AFP and CA125 assays and abdominal US scan showed no evidence of tumor recurrence in the woman. The baby had normal physical and neurological development.

## Discussion

The incidence of pregnancy complicated by ovarian cancer is low. Among the ovarian cancers reported in one study, 47.8 % were malignant germ cell tumors, 21.7 % were borderline malignant tumors, 17.4 % were invasive epithelial tumors, and 13.1 % were sex cord stromal tumors [4]. In all, 73.9 % of those patients were diagnosed in stage I disease and had complete remissions [4]. Another literature review of 41 cases of maternal epithelial ovarian cancer reported between 1958 and 2007 revealed that the mean age of the patients at the first visit was 32.6 years (range 23−46 years), with 69 % >30 years, 36 % >35 years, and 28 % >40 years [5].

Primary ovarian carcinoma occurs most commonly in women of low parity during the latter half of their reproductive years and remains the fifth most common cause of cancer-related death for women in the United States [5]. A study showed that of 28,165 patients diagnosed with epithelial ovarian cancer, 400 were <30 years (very young), 11,601 were 30−60 (young), and 16,164 were >60 (older) years of age. Moreover, very young women usually had a significant survival advantage over the young and older groups with 5-year disease-specific survival estimates at 78.8 % versus 58.8 and 35.3 %, respectively [7].We reported here a 25-year-old woman with ovarian cystadenocarcinoma who underwent emergent surgery because of torsion of a mass during the first trimester. Her case posed great challenges for the multidisciplinary team responsible for her care owing to postoperative confirmation of a malignant tumor without staging and the presence of a first trimester pregnancy. At this stage, the embryo is sensitive to chemotherapy including cisplatin, carboplatin, toxtocile, and bleomycin [8]. It was recommended that she undergo unilateral adnexectomy if it was a stage IA tumor or bilateral adnexectomy if it was a stage IB tumor; in the latter case (grade B), peritoneal cytology and complete abdominopelvic exploration would be added [9]. The patient insisted on continuing the pregnancy, and she did not undergo staging surgery or chemotherapy. If she had undergone surgery, it would have had adverse effects on the embryo; and chemotherapy was contraindicated during the first trimester [4–6].

It has been recommended in previous reports that pregnant women with ovarian malignancy should be treated in the same way as nonpregnant women [5, 10], with similar prognoses for the nonpregnant and pregnant women at the same cancer stage [5, 9–13]. When considering surgical intervention during the first trimester, we have to face the risk of spontaneous miscarriage following laparotomy. The second trimester is generally regarded as the best time for surgical intervention because the risk of miscarriage is lower [10, 11].

In 2009, Palmer et al. [5] reported that since 1968 only three women had undergone surgery during the first trimester. One of them experienced subsequent termination of the pregnancy during the second trimester. Although no adverse event following surgery was reported during the first trimester, the number was too low to draw a general conclusion. Our case is therefore a useful contribution to the treatment of maternal ovarian cancer during the first trimester.

Nonpregnant patients with stage I ovarian cancer rarely have recurrence after unilateral salpingo-oophorectomy, and they have a good prognosis, with 5-year survival rates >90 % [14–17]. No significant difference in overall outcome was found between patients with incomplete staging and those with complete staging [18]. Nonpregnant patients with advanced ovarian cancer were prone to relapse, with 5-year survival rates <30 % [17–20].

Our patient was the first reported case of a recurrent tumor 32 weeks after salpingo-oophorectomy at week 6^+5^ of gestation. Cytoreductive surgery proved that the isolated complex mass located between the bladder and uterus was metastatic ovarian adenocarcinoma; the other organs including pelvic and para-aortic lymph nodes were negative for malignancy. At 47 months after the last chemotherapy was given, there was no evidence of recurrence clinically or radiologically. This case was different from those in previous reports in which patients with early-stage ovarian cancer were more prone to relapse but had a better prognosis than nonpregnant women with late-stage ovarian cancer. The metastatic tumors were usually disseminated from advanced ovarian cancers in nonpregnant women. Our case was an isolated tumor in stage C. It is important to consider the impact of the cancer on the pregnancy and, conversely, the impact of the pregnancy on the cancer.

Previous reports noted that tumor markers are of limited value for substantiating a diagnosis during pregnancy because the serum CA125 levels may be elevated in normal pregnancy, usually peaking in the first trimester and returning to normal range (<65 U/ml) in the second trimester [5, 21–23]. In our case, the serum CA125 level was 121 U/ml at an early stage and dropped to 93 U/ml 3 days after surgery. When the tumor relapsed, the serum CA125 levels rose to 381 U/ml and again dropped to normal after cytoreductive surgery following three cycles of chemotherapy. The fluctuation of CA125 in this case was similar to that in nonpregnant women with epithelial ovarian cancer, suggesting that CA125 may provide some information when screening, diagnosing, and monitoring some epithelial ovarian cancers during pregnancy.

In summary, the treatment of epithelial ovarian carcinoma during pregnancy remains a challenge because of the paucity of data and the impact on the fetus. It is important to consider the reciprocal effects on the cancer and the pregnancy. Intensive follow-up of the patient is necessary because the tumor is prone to recurrence. More cases need to be analyzed to obtain a clear picture of the long-term survival rate.
